# Central and peripheral contributions to dynamic changes in nucleus accumbens glucose induced by intravenous cocaine

**DOI:** 10.3389/fnins.2015.00042

**Published:** 2015-02-12

**Authors:** Ken T. Wakabayashi, Eugene A. Kiyatkin

**Affiliations:** Behavioral Neuroscience Branch, National Institute on Drug Abuse – Intramural Research Program, National Institutes of Health, DHHSBaltimore, MD, USA

**Keywords:** high-speed amperometry, enzyme-based glucose sensors, nucleus accumbens, metabolism, cerebral blood flow, neural activity

## Abstract

The pattern of neural, physiological and behavioral effects induced by cocaine is consistent with metabolic neural activation, yet direct attempts to evaluate central metabolic effects of this drug have produced controversial results. Here, we used enzyme-based glucose sensors coupled with high-speed amperometry in freely moving rats to examine how intravenous cocaine at a behaviorally active dose affects extracellular glucose levels in the nucleus accumbens (NAc), a critical structure within the motivation-reinforcement circuit. In drug-naive rats, cocaine induced a bimodal increase in glucose, with the first, ultra-fast phasic rise appearing during the injection (latency 6–8 s; ~50 μM or ~5% of baseline) followed by a larger, more prolonged tonic elevation (~100 μM or 10% of baseline, peak ~15 min). While the rapid, phasic component of the glucose response remained stable following subsequent cocaine injections, the tonic component progressively decreased. Cocaine-methiodide, cocaine's peripherally acting analog, induced an equally rapid and strong initial glucose rise, indicating cocaine's action on peripheral neural substrates as its cause. However, this analog did not induce increases in either locomotion or tonic glucose, suggesting direct central mediation of these cocaine effects. Under systemic pharmacological blockade of dopamine transmission, both phasic and tonic components of the cocaine-induced glucose response were only slightly reduced, suggesting a significant role of non-dopamine mechanisms in cocaine-induced accumbal glucose influx. Hence, intravenous cocaine induces rapid, strong inflow of glucose into NAc extracellular space by involving both peripheral and central, non-dopamine drug actions, thus preventing a possible deficit resulting from enhanced glucose use by brain cells.

## Introduction

While recently much attention has been focused on the mechanisms underlying cocaine's addictive properties, cocaine is a psychoactive drug that also stimulates motor activity (Camp et al., 1994), increases arterial blood pressure (Poon and Van Den Buuse, 1998), enhances whole-body and cerebral oxygen consumption (Ceolin et al., 2007), and elevates brain and body temperatures (Kiyatkin and Brown, 2005). While all these effects suggest metabolic neural activation, direct attempts to evaluate the central metabolic effects of cocaine with deoxyglucose radiography and positron emission tomography (PET) have produced conflicting results. Some data indicate that cocaine increases glucose utilization in the striatum and related structures (London et al., 1986; Porrino et al., 1988; Thomas et al., 1996), suggesting metabolic activation, while others have reported decreases in cellular glucose consumption (London et al., 1990; Lyons et al., 1996; Porrino et al., 2002; Thanos et al., 2008), consistent with metabolic inhibition.

Direct measurement of cocaine-induced fluctuations in extracellular glucose could serve as an important tool to resolve this apparent discrepancy and clarify how this critical metabolic parameter is affected by cocaine. In contrast to deoxyglucose measurements, which characterize glucose uptake by brain cells generally at single integrated time points (Hodgkin, 1967; Ritchie, 1973; Sokoloff, 1999), extracellular glucose levels depend upon two opposing variables: its metabolic use by brain cells and its intra-brain entry from the arterial blood by a gradient-dependent facilitated diffusion via the GLUT-1 transporter (Fellows and Boutelle, 1993; Silver and Erecinska, 1994; De Vries et al., 2003). Using enzyme-based glucose biosensors with high-speed amperometry, we recently showed that glucose levels in the nucleus accumbens (NAc), a critical structure for sensorimotor integration and reinforcement (Mogenson et al., 1980; Wise and Bozarth, 1987; Di Chiara, 2002), phasically increase during exposure to sensory stimuli of different modality (Kiyatkin and Lenoir, 2012). Since sensory stimuli excite accumbal neurons (Kiyatkin and Rebec, 1999), these phasic glucose increases appear to reflect its rapid, neural activity-regulated entry from the arterial blood. Intravenous (iv) cocaine, by acting on ionic channels on the afferents of visceral sensory nerves, also induces transient activation of accumbal neurons (Kiyatkin and Brown, 2007) and phasic NAc glutamate release (Wakabayashi and Kiyatkin, 2014). Therefore, a similar peripherally triggered central mechanism could be engaged by cocaine, inducing rapid glucose entry into brain tissue.

This study was designed to test this hypothesis by direct monitoring of NAc extracellular glucose levels using enzyme-based glucose biosensors coupled with high-speed amperometry in three groups of freely moving rats. First, we examined how iv cocaine at a low, behaviorally relevant dose affects NAc glucose levels following four repeated injections during 1 day-long recording session. Second, using the same protocol, we compared these responses to those induced by cocaine-methiodide, a peripherally acting cocaine analog (Shriver and Long, 1971; Hemby et al., 1994; Wise et al., 2008). Lastly, to examine the contribution of dopamine (DA) to cocaine-induced NAc glucose responses, they were compared to those conducted during full DA receptor blockade induced by a mixture of D1- and D2-selective DA antagonists.

## Materials and methods

### Animals and surgeries

Data from 34 male Long-Evans rats (Charles River Laboratories) weighing 460 ± 40 g at the time of testing were used in this study. Rats were individually housed in a climate-controlled animal colony maintained on a 12-12 light-dark cycle (lights on at 07:00), with food and water available *ad libitum*. All procedures were approved by the NIDA-IRP Animal Care and Use Committee and complied with the Guide for the Care and Use of Laboratory Animals (NIH, Publication 865-23).

Under general anesthesia (Equithesin 0.33 ml/100 g, ip), rats were implanted with a BASi cannula (Bioanalytical Systems, Inc.; West Lafayette, IN) for future insertions of the sensor in the medial sector of the nucleus accumbens (NAc shell). The target coordinates were: AP + 1.2 mm, ML ± 0.8 mm and DV 7.3 mm, according to the stereotaxic atlas of Paxinos and Watson (1998). The guide cannula hub was fixed to the skull with a head mount constructed from dental acrylic that was secured using three stainless steel bone screws. During the same surgical procedure, rats were also implanted with a chronic jugular catheter, which ran subcutaneously to the head mount and was secured to the same head assembly. Rats were allowed a minimum of 4 days of post-operative recovery; jugular catheters were flushed daily with 0.2 ml heparinized saline (10 units/ml) to maintain patency.

### Fixed-potential amperometry with enzyme-based electrochemical sensors

Commercially produced glucose oxidase-based biosensors (Pinnacle Technology, Inc.) coupled with fixed-potential amperometry have been extensively used in our previous studies (Kiyatkin and Lenoir, 2012; Kiyatkin et al., 2013). These reports describe in detail issues regarding the sensitivity/selectivity and *in vitro* and *in vivo* performance of these sensors. We also established the importance of control recordings with enzyme-free null sensors to minimize the contribution of non-specific chemical and physical influences that affect measurements made with enzyme-based biosensors in freely moving animals. Since enzyme-free null sensors are identically constructed, have a comparable sensitivity to major anionic (i.e., ascorbate) and cationic (i.e., DA) contaminants, are similarly temperature-sensitive, and show a similar downward drift in current during a long-term *in vitro* and *in vivo* recording, their use reduces the contribution of various chemical and physical interferents to reveal dynamic fluctuations in extracellular glucose.

Both glucose and null sensors are prepared from Pt-Ir wire of 180 μm diameter, with a sensing cavity of ~1 mm length on its tip. The active electrode in both types of sensors is incorporated with an integrated Ag/AgCl reference electrode. On the active surface, glucose oxidase converts glucose to glucono-1, 5-lactone and hydrogen peroxide (H_2_O_2_), which is detected as an amperometric oxidation current generated by a +0.6 V applied potential (Hu and Wilson, 1997). The potential contribution of ascorbic acid to the measured current is competitively reduced by co-localizing ascorbic acid oxidase enzymes on the active surface of the sensor. This enzyme converts ascorbic acid to non-electroactive dehydroascorbate and water. In addition, a negatively charged Nafion polymer layer under the enzyme layer serves to exclude endogenous anionic compounds (Hu and Wilson, 1997). Null sensors are prepared identically to glucose sensors except for the absence of glucose oxidase.

Both types of sensors were calibrated immediately before and after each *in vivo* experiment. *In vitro* calibrations were conducted in PBS (pH 7.3) by incrementally increasing the concentration of glucose (Sigma-Aldrich) from 0 to 0.5, 1.0, and 1.5 mM followed by a single addition of ascorbate (25 μM). Within this physiological range (Fellows and Boutelle, 1993; McNay et al., 2001), glucose sensors used in this study produced incremental linear current increases. Mean sensitivity to glucose was 2.62 ± 0.28 nA/0.5 nM at 22–23°C and 5.12 nA at 37°C. Glucose sensors showed low sensitivity to ascorbate (0.12 ± 0.02 nA/25 μM at 22–23°C) and, as showed previously, they were low sensitive to DA at its physiological levels (5–50 pA/10–100 nM). Glucose sensors remained equally sensitive to glucose and selective against ascorbate during post-recording *in vitro* calibrations (2.23 ± 0.22 nA/0.5 mM and 0.10 nA ± 0.03/25 μM, respectively). Consistent with their design, null sensors generated no oxidation current following repeated applications of glucose (0–2 mM) and showed equally small current response to application of ascorbate (0.06 ± 0.03 nA/25 μM). Differences in basal currents detected *in vivo* by both types of sensors were used for calculating basal levels of glucose in the NAc and their possible changes during the recording sessions.

### Experimental protocol

*In vivo* electrochemical procedures occurred during the day (9:00–18:00) in an electrically insulated chamber (38 × 47 × 47 cm) under continuous dim illumination (20 W red light bulb), with a room wide air filter fan providing background noise. The cage was equipped with four infrared motion detectors (Med Associates, Burlington, VT, USA), which were used to monitor rat locomotion. Prior to recording, rats were habituated to the testing environment for a minimum of 6 h per day for 3 consecutive days.

At the beginning of each experimental session, rats were minimally anesthetized (>2 min) with isoflurane and a calibrated sensor (either glucose or null) was inserted into the brain through the guide cannula. The sensor was connected to the potentiostat (Model 3104, Pinnacle Technology) via an electrically shielded flexible cable and a multi-channel electrical swivel. Additionally, the injection port of the jugular catheter on the head mount was connected to two plastic catheter extensions that allowed stress- and cue-free delivery of saline and the tested drug from outside the cage, thus minimizing possible detection of the iv drug injection by the rat. Testing began ~140 min after insertion of the sensor when the baseline currents relatively stabilized.

Each rat was recorded during one daily session with either glucose or null sensor. Prior to all experiments, rats were subjected to three control stimuli presented 15 min apart. These stimuli were: a brief auditory stimulus (75 dB, 0.25 s), presentation of a novel object (a small glass beaker manually introduced and later removed from the cage) for 1 min, and one or two saline injections (0.2 ml over 20 s) delivered via a dedicated catheter extension from outside the cage, thus excluding any stress or cue associated with its delivery. These control tests were important for assessing *in vivo* sensor performance, evaluating the contribution of sensory input and arousal to changes in [glucose] during cocaine exposure, and to ascertain the response to the procedure of injection. Then, three sequential experiments were conducted.

In Experiment I (*n* = 13 rats; 7 with glucose and 6 with null sensors), we examined changes in glucose levels ([glucose]) induced by four repeated cocaine injections within the same 8–9-h recording session. Cocaine HCl (1 mg/kg in 0.2 ml saline over 20 s) was iv injected with 90-min inter-injection intervals. Similar to saline injection, cocaine was delivered via separate catheter extension from outside the cage, thus excluding any stress or cue associated with the injection. This dose of cocaine is within the range for the development and maintenance of self-administration in rats (Pickens and Thompson, 1968; De Wit and Stewart, 1981; Kiyatkin and Stein, 1995; Wise et al., 1995; Kiyatkin and Brown, 2003) and induces clear behavioral, physiological, and neurochemical effects (Wise et al., 1995, 2008; Brown and Kiyatkin, 2005; Wakabayashi and Kiyatkin, 2014). Cocaine at this dose was also used in our previous studies, thus allowing us to compare different sets of behavioral, physiological and neurochemical data. The interval between injections is about 10-fold longer than the half-life of cocaine with iv administration in freely moving rats (Tsibulsky and Norman, 1999) and is sufficiently enough for all basic physiological parameters to return to baseline. At the end of each session, rats were iv injected with Equithesin (0.8 ml by iv injection over 2 min) to induce general anesthesia. Then, the rat was disconnected from the potentiostat and the sensor was removed for post-recording calibrations.

Experiment II (*n* = 11 rats; 6 with glucose and 5 with null sensors) was conducted with an identical protocol but, instead of cocaine, rats received four injections of cocaine-methiodide (1.33 mg/kg in 0.2 ml saline over 20 s) delivered with the same, 90-min inter-injection intervals. The dose of cocaine-methiodide is equimolar to that of cocaine HCl and this drug at this dose induces a clear behavioral response during the injection duration, rapid and robust changes in EEG and EMG (Kiyatkin and Smirnov, 2010), increases in NAc glutamate (Wakabayashi and Kiyatkin, 2014), and modest increases in brain and body temperature (Brown and Kiyatkin, 2006).

In Experiment III (*n* = 10 rats; 7 with glucose and 3 with null sensors), we examined the changes in [glucose] induced by four cocaine injections conducted during full pharmacological blockade of DA transmission induced by a mixture of selective D1-like (SCH 23390, 0.4 mg/kg) and D2-like (eticlopride, 0.4 mg/kg) receptor antagonists. Under a modified protocol similar to experiments I and II, rats received subcutaneously a mixture of these drugs (0.4 ml) 20 min prior to the first cocaine injection, with additional maintenance doses of 0.2 mg/kg 15–20 min prior to each subsequent cocaine injection. These drugs are highly selective toward D1 and D2 receptors (relative D1:D2 affinity, SCH = 2500:1 and ETI = 1:514,000; Neve and Neve, 1997) and at this combined dose significantly attenuate striatal neuronal responses to DA for at least 90 min post-injection (Kiyatkin and Rebec, 1999) and fully block cocaine-induced locomotor responses (Kiyatkin, 2008). Relatively large doses of DA antagonists and a within-session drug boosting were used to maintain full blockade of DA transmission during the session.

### Histology

Under deep anesthesia with Equithesin, rats were transcardially perfused with room-temperature PBS (pH 7.4) followed by 10% formalin. Brains were sectioned on a cryostat to a thickness of 45 μm. The location of the sensors was verified using the stereotaxic atlas of Paxinos and Watson (1998).

### Data analysis

Electrochemical data were sampled at 1 Hz (i.e., mean current over 1 s) using the PAL software (Version 1.5.0, Pinnacle Technologies) and analyzed using two time resolutions. Slow changes in electrochemical currents were analyzed with 1-min quantification bins using an analysis window of 5 min before and 60 min after each iv drug injection. Rapid current changes were analyzed with 2-s bins for 30 s before and 180 s after control stimuli presentation, saline, and drug injections. Since the baseline currents slightly varied in amplitude between individual glucose electrodes, and both glucose and null sensors showed current changes following a stimulus presentation or drug injection, absolute current changes were transformed into relative changes taking a basal value before each event (30 s for slow and 8 s for rapid time-course analyses) as 0 nA. Current differentials (changes generated by each glucose sensor minus mean changes generated by null sensors) were then calculated to reduce the influence of extraneous physical and chemical contributors to the glucose current. These current differences were then transformed into glucose concentration (μM) based on sensor sensitivity determined during pre-recording *in vitro* calibrations and adjusted by the temperature coefficient (95.6%) determined in previous analytical studies (Kiyatkin et al., 2013) and confirmed for selected sensors in this study. Locomotor activity was quantified as the number of infrared beam breaks per minute. These data were used to determine the relationships between changes in glucose and drug-induced motor activation.

Statistical data analyses included three stages. First, we determined whether relative changes in currents detected by the glucose and null sensors differed from each other, using two-way repeated measures (RM) ANOVA. Since both glucose and glucose-null currents were analyzed as a change from 0 nA baseline, the length of the drug effect was determined as the average duration when either the two currents were different (significant glucose vs. null current main effect) or when the glucose current was changing with respect to the null current (significant Current × Time interaction). Second, if a significant effect was found, One-Way RM ANOVAs were conducted on calculated [glucose] values to find time periods where there was a significant post-injection main effect and individual bins were compared with respect to baseline using a Fisher *post-hoc* test. One-Way RM ANOVA was also used for evaluating statistical significance of changes in locomotion. Fisher *post-hoc* tests were used for pair-wise comparisons, and the latency of the glucose response was determined based on the first data point significantly different from baseline (*p* < 0.05). Third, differences in total effects of repeated drug injections for both [glucose] and locomotion were expressed as the area under the curve (1-min bins [glucose] and locomotion: 20 min post-injection; 2-s bins [glucose]: 30 s post-injection); mean values of this parameter were analyzed for each drug using One-Way RM ANOVA with subsequent Fisher *post-hoc* tests. For clarity, statistical details are presented in the figure legends.

## Results

By comparing basal electrochemical currents recorded by glucose and null sensors during *in vivo* recordings and transferring their differences into concentration values, we estimated that basal [glucose] in the NAc shell (~2 h after sensor insertion in the brain) was 702 ± 40 μM (*SD* = 171 μM). These values remained relatively stable during an ~8-h recording session, slightly decreasing by its end (585 ± 37 μM; *SD* = 158 μM). These values are consistent with previous estimates obtained with no-net-flux microdialysis and electrochemistry in the striatum of awake rats (0.47 mM; Fellows et al., 1992; 0.71 mM, McNay and Gold, 1999; 0.39 mM, Lowry et al., 1998) as well as NAc glucose levels determined in our previous study (540 μM; Kiyatkin and Lenoir, 2012).

### Cocaine induces rapid and strong bi-modal increases in NAc extracellular [glucose]

**Figure 1:**
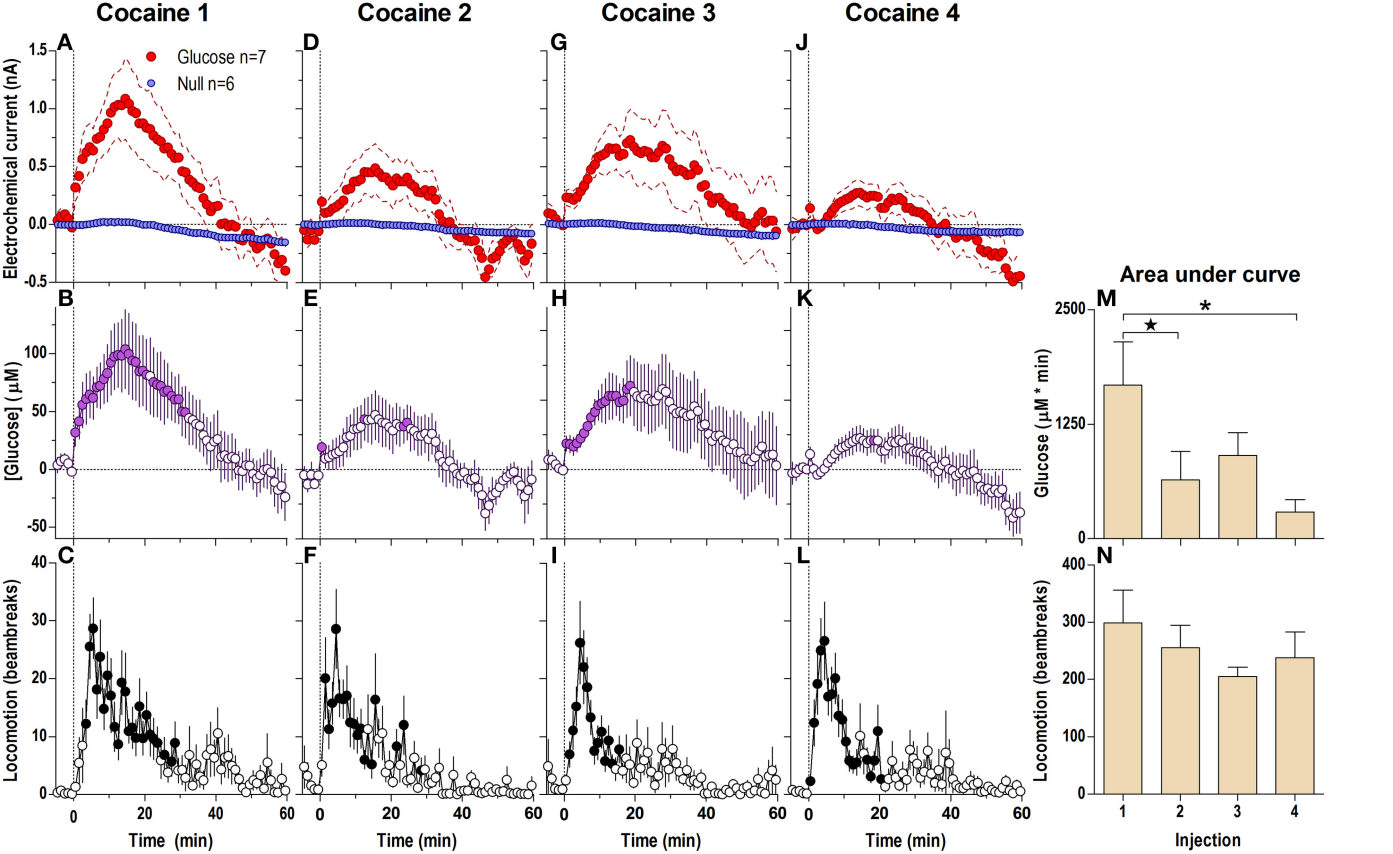
**Relative changes in NAc [glucose] induced by cocaine injections assessed at low temporal resolution (1-min bins)**. Top graphs **(A,D,G,J)** show mean ± SEM changes in relative currents (nA) detected by Glucose and Null sensors. Middle graphs **(B,E,H,K)** show mean ± SEM changes in [glucose] (μM) as a difference between active and null sensors. Bottom graphs **(C,F,I,L)** show changes in locomotor activity (mean ± SEM; counts/min). Vertical hatched lines (at 0 min) marked the onset of 20-s cocaine injection. Horizontal dotted lines show basal levels (= 0 nA and μM). The difference in current dynamics between active and null sensors was significant (*p* < 0.05) for the entire 60-min duration after each cocaine injection [Two-Way repeated measures (RM) ANOVA; Current × Time interaction *F*_(6, 660)_ = 5.94, 3.03, 1.94, and 4.72, all *p* < 0.05 for injections 1–4, respectively]. Concentration change was also significant for each cocaine injection [*F*_(60, 360)_ = 7.07, 3.63, 2.30, and 5.61, all *P* < 0.05 respectively]. Individual concentration values significantly different from baseline (Fisher test) are shown as filled symbols. Cocaine induced significant locomotor activation after each injection [*F*_(12, 732)_ = 4.14, 5.45, 4.61, 5.20 for injections 1–4, respectively; *p* < 0.05]. Right panels **(M,N)** show differences in mean ± SEM values of glucose and locomotor responses induced by cocaine injections as assessed by area under the curve. The effect of injection number was significant for [glucose] [One-Way RM ANOVA: *F*_(3, 18)_ = 4.94, *p* < 0.05] but not significant for locomotion. Asterisks and star show significant between-injection differences.

When analyzed at the 1-min time-scale, the initial cocaine injection in a drug-naive rat induced a significantly different current response in the glucose and null sensors [Current × Time interaction; *F*_(6, 660)_ = 5.94; *p* < 0.05] for the entire 60-min analysis duration (Figure 1A), revealing a rapid, significant [glucose] increase from the first minute post-injection [Figure 1B; *F*_(60, 360)_ = 7.07, *p* < 0.05]. This increase peaked at 15–20 min (~110 μM or ~11% of baseline) followed by a slow return to baseline at ~45 min post-injection. Importantly, the largest rate of [glucose] increase occurred during the first min post-injection. Cocaine also induced modest locomotor activation (Figure 1C) for ~20–30 min post-injection [*F*_(12, 732)_ = 4.14, *p* < 0.05]. While the rise in [glucose] resulted primarily from current changes detected by glucose sensors, cocaine also induced a tonic increase in currents detected by null currents; this latter change was much smaller than that recorded by glucose sensors.

Each subsequent cocaine injection also induced significant differences between glucose and null currents for the entire 60-min analysis interval [Figures 1D,G,J; Current × Time interaction *F*_(6, 600)_ = 3.03, 1.94, and 4.72, respectively; *p* < 0.05]. These differences revealed increases in [glucose] with each injection, with the largest rate of increases occurring during the first post-injection minute (Figures 1E,H,K). However, the magnitude and duration of this slow [glucose] increase became weaker with each subsequent injection [Figure 1M; *F*_(3, 18)_ = 4.94, *p* < 0.05]. In contrast, there were no significant differences in cocaine-induced locomotor activation with each of the four cocaine injections (Figure 1N), although there was a tendency for a more rapid onset (Figures 1C,F,I,L).

**Figure 2:**
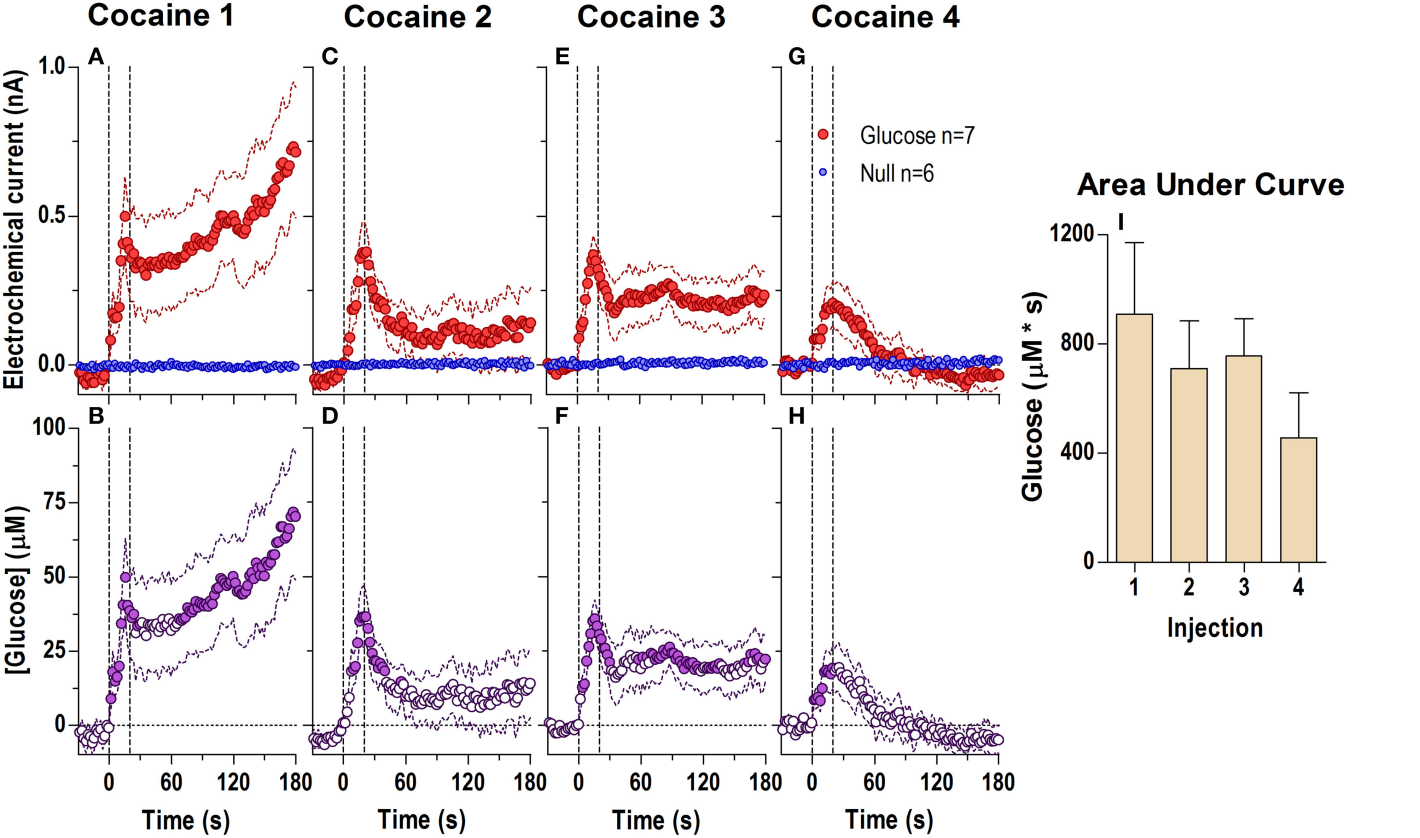
**Relative changes in NAc [glucose] induced by cocaine injections assessed at high temporal resolution (2-s bins)**. Top graphs **(A,C,E,G)** show mean ± SEM changes in relative currents (nA) detected by Glucose and Null sensors. Bottom graphs **(B,D,F,H)** show mean ± SEM changes in [glucose] (μM) as a difference between active and null sensors. Two vertical hatched lines (at 0 and 20) marked the onset and offset injection. Horizontal dotted lines show basal levels (= 0 nA and μM). After cocaine injections, the Glucose and Null currents differed significantly (**A** 1: Glucose/Null [180 s, *F*_(1, 11)_ = 6.97], interaction [180 s, *F*_(90, 990)_ = 2.88]; **C** 2: Interaction [180 s, *F*_(90, 990)_ = 1.44]; **E** 3: Glucose/Null [180 s, *F*_(1, 11)_ = 8.31], interaction [153.5 s, *F*_(77, 847)_ = 1.31]; **G** 4: Glucose/Null [47.5 s, *F*_(1, 11)_ = 4.69], interaction [180s, *F*_(90, 990)_ = 3.58], all *p* < 0.05), resulting in a significant [glucose] change for each cocaine injection during the entire analysis window [*F*_(6, 546)_ = 3.47, 1.69, 1.37, and 4.31, all *p* < 0.05]. Concentration values significantly different from baseline (Fisher test) are shown as filled symbols. Right panel **(I)** shows mean ± SEM values of glucose responses induced by cocaine injections assessed by area under the curve for 30 s after the injection onset (n.s.).

When analyzed at the second scale (Figure 2; 2-s bins, 180-s analysis window), we found significant differences between glucose and null currents after all cocaine injections (Figures 2A,C,E,G; see statistical details in figure legends), indicating a rapid increase in [glucose] during each drug injection (Figures 2B,D,F,H). This rise peaked near the end of the injection (20–50 μM or a 3–7% increase) and began to fall thereafter. Immediately after the first injection (Figure 2A), this post-injection decrease was minimal before the onset of the second, slower increase clearly seen in the longer 60-min analysis window (see Figure 1A). However, during each subsequent injection the onset of this second rise was weaker and delayed, revealing a gap distinguishing the rapid and slow components of the glucose response to cocaine (Figures 2D,F,H). The rapid initial rise was relatively stable after each cocaine injection, but showed a tendency to decrease in magnitude and duration (Figure 2I).

The cocaine-induced changes in [glucose] were not related to the procedure of drug injection. Our control tests revealed that iv injection of saline did not induce any increase in electrochemical currents (see **Figure 7** below). In contrast to cocaine, currents detected by the glucose sensors slightly decreased during a saline injection, whereas null currents remained relatively stable [**Figure 7A**; Current × Time interaction *F*_(90, 2430)_ = 1.94, *p* < 0.05], indicating a weak but significant drop in [glucose] [**Figure 7D**; ~10 μM or ~1% vs. baseline, *F*_(18, 1620)_ = 3.77, *p* < 0.05]. Saline injection had no effects on locomotor activity.

### BBB-impermeable cocaine-methiodide induces equally rapid and strong increases in NAc extracellular [glucose] but no tonic glucose elevation and no motor activation

Previously, we have found that cocaine-methiodide, a peripherally acting cocaine analog, mimics cocaine in its ability to induce cortical EEG desynchronization and EMG activation (Kiyatkin and Smirnov, 2010), excite accumbal and ventral tegmental area neurons (Kiyatkin and Brown, 2007; Brown and Kiyatkin, 2008) and induce rapid NAc but not slow glutamate release (Wakabayashi and Kiyatkin, 2014). The ultra-rapid dynamics of cocaine-induced changes in [glucose] and its similarity with glucose responses induced by sensory stimuli (Kiyatkin and Lenoir, 2012 and **Figure 7** below) suggest a peripherally triggered neural mechanism as its origin. To test this hypothesis, we examined how cocaine-methiodide impacts NAc extracellular [glucose] and how these responses differ from those induced by regular cocaine, which acts in both the brain and the periphery.

**Figure 3:**
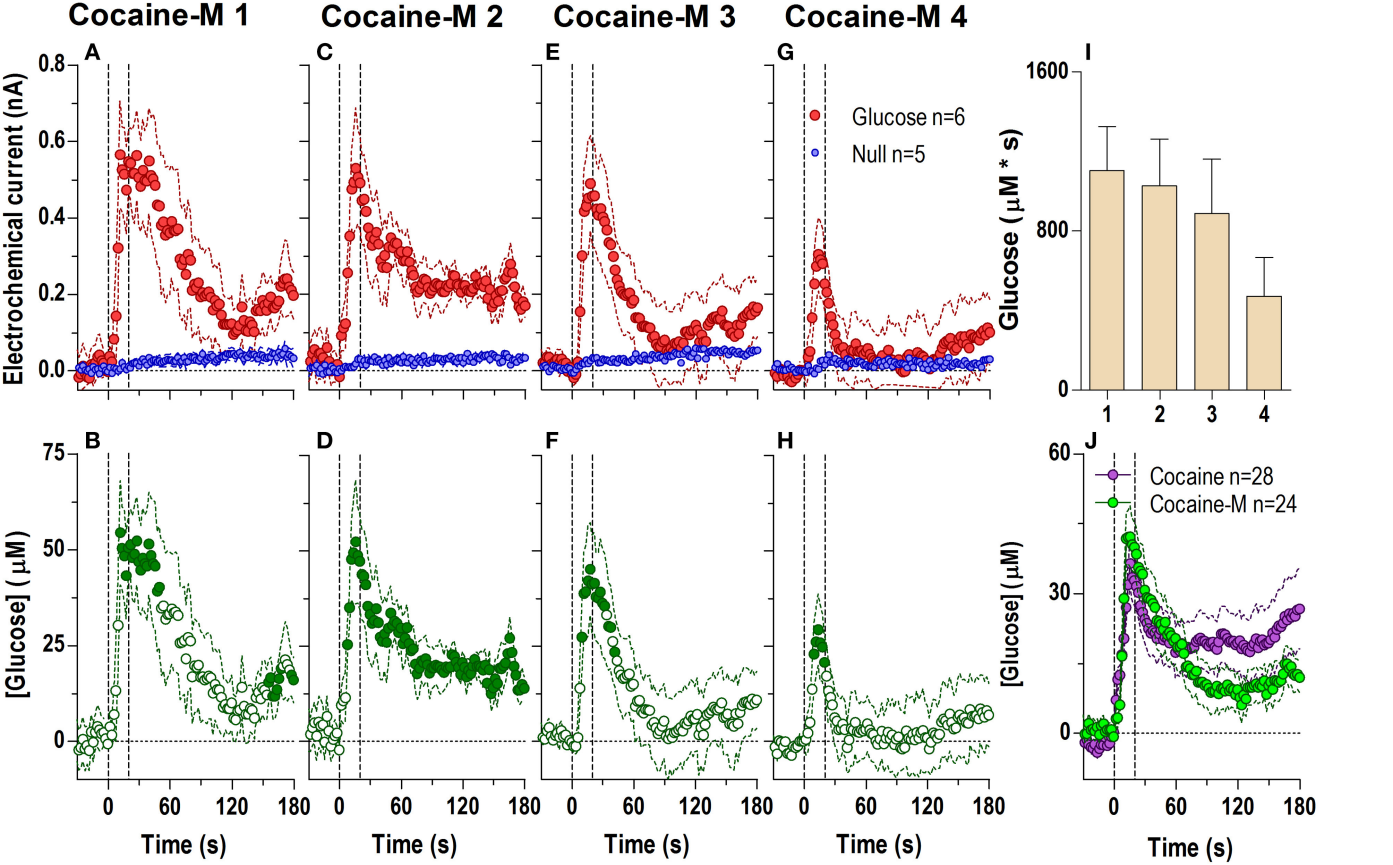
**Relative changes in NAc [glucose] induced by cocaine-methiodide injections assessed at high temporal resolution (2-s bins)**. Top graphs **(A,C,E,G)** show mean ± SEM changes in relative currents (nA) detected by Glucose and Null sensors. Bottom graphs **(B,D,F,H)** show mean ± SEM changes in [glucose] (μM) as a difference between Glucose and Null sensors. Two vertical hatched lines (at 0 and 20) marked the onset and offset of the injection. Horizontal dotted lines show basal levels (= 0 nA and μM). The difference in current dynamics between active and null sensors was significant (*p* < 0.05) for the entire 180-s duration after each cocaine injection (**A** 1: Glucose/Null [*F*_(1, 9)_ = 6.69], interaction [*F*_(91, 819)_ = 3.60]; **C** 2: Glucose/Null [*F*_(1, 9)_ = 26.6], interaction [*F*_(91, 819)_ = 2.23]; **E** 3: Glucose/Null [*F*_(1, 9)_ = 1.85], interaction [*F*_(91, 819)_ = 4.03]; **G** 4: interaction [*F*_(91, 819)_ = 2.33], all *p* < 0.05]. Concentration change was also significant for each cocaine injection for the entire analysis window [*F*_(5, 445)_ = 4.28, 2.74, 5.01, and 2.95, all *p* < 0.05]. Individual values significantly different from baseline (Fisher test) are shown as filled symbols. Right panel **(I)** shows mean ± SEM v glucose responses assessed by area under the curve (n.s.). Right panel **(J)** compares the mean glucose response between the cocaine and cocaine-methiodide group; no significant differences found.

When injected at an equimolar dose (Figure 3) cocaine-methiodide induced highly rapid increases in the glucose current while having a minimal influence on the null current when all four injections were analyzed at second-scale resolution (Figures 3A,C,E,G). The differences between these currents revealed significant increases in [glucose] during and immediately after each drug injection (Figures 3B,D,F,H). However, these changes differed from those seen with regular cocaine. While the rapid effects of cocaine-methiodide were very similar to cocaine in their time-course, magnitude and tendency to show a slight tolerance for ~30 s after the injection onset (Figure 3I), cocaine-methiodide always induced an unimodal, phasic increases with no evidence of a second, tonic rise seen most clearly during the first cocaine injection. When compared as an average for all injections in cocaine and cocaine-methiode groups (Figure 3J), the time-course of changes was surprisingly similar with no statistical differences between groups.

**Figure 4:**
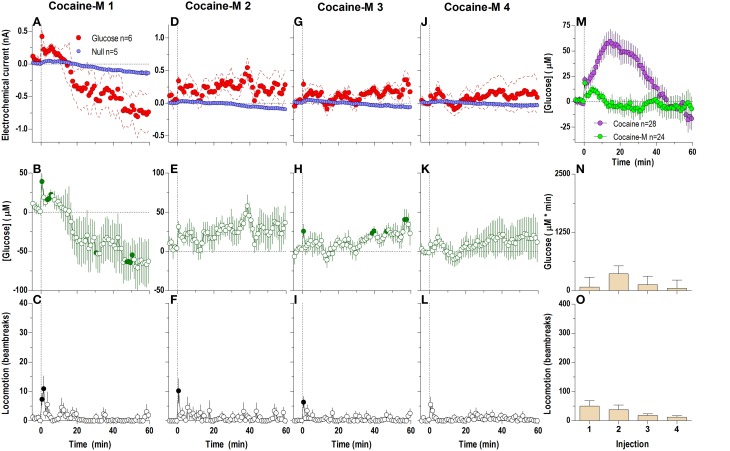
**Relative changes in NAc [glucose] induced by injections of cocaine-methiodide assessed at low temporal resolution (1-min bins)**. Top graphs **(A,D,G,J)** show mean ± SEM changes in relative currents (nA) detected by Glucose and Null sensors. Middle graphs **(B,E,H,K)** show mean ± SEM changes in [glucose] (μM) as a difference between Glucose and Null sensors. Bottom graphs **(C,F,I,L)** show changes in locomotor activity (mean ± SEM; counts/min). Vertical hatched lines (at 0 min) marked the onset of 20-s drug injection. Horizontal dotted lines show basal levels (= 0 nA and μM). There were significant differences in Glucose and Null currents for the first three injections (**A** 1: interaction [59.5 min, *F*_(60, 540)_ = 2.64]; **D** 2: Glucose/Null [10.5 min *F*_(1, 9)_ = 5.72], interaction [3.5 min *F*_(4, 36)_ = 2.91]; **G** 3: Glucose/Null [59.5 min *F*_(1, 9)_ = 6.08], interaction [2.5 min *F*_(3, 27)_ = 3.08], all *p* < 0.05), and no changes for injection 4. This resulted in significant changes in glucose concentration after the first [*F*_(60, 300)_ = 3.83] and third injection [*F*_(60, 300)_ = 1.49, both *p* < 0.05] for the entire analysis window. Individual concentration values significantly different from baseline (Fisher test) are shown as filled symbols. **(M)** shows significant differences in mean ± SEM glucose responses to cocaine and cocaine methiodide [mean of 4 injections; Main effect *F*_(1, 50)_ = 5.89, Drug × Time interaction *F*_(60, 3000)_ = 8.20, both *p* < 0.05]. **(N,O)** show mean ± SEM glucose and locomotor responses induced by cocaine-methiodide injections as assessed by area under the curve (both n.s.).

The lack of a tonic effect can be seen even more clearly at the 1-min time scale (Figure 4), where cocaine-methiodide induced changes in the glucose current relative to the null current for only the first three injections (Figures 4A,D,G,J), and only showed relatively clear changes in [glucose] during the first injection [Figure 4B; *F*_(60, 300)_ = 3.83 *p* < 0.05]. Unlike regular cocaine, this increase in [glucose] in this case was transient (13–15 min) and was followed by a strong decrease below the pre-injection baseline. The lack of a tonic rise contributed to an overall much lower [glucose] response assessed as an area under the curve (Figure 4N). When the averages for all injections were compared for cocaine and cocaine-methiodide groups (Figure 4M), both drugs induced identical NAc [glucose] changes for the first post-injection minute, and thereafter these changes drastically differed from each other. Consistent with our previous findings (Brown and Kiyatkin, 2006; Wakabayashi and Kiyatkin, 2014), cocaine-methiodide induced only minimal increases in locomotion during and immediately after the injection (Figures 4C,F,I,L,O).

### Both components of the NAc glucose response persist but become weaker during pharmacological blockade of DA transmission

While inhibition of DA reuptake and a subsequent increase in DA levels is commonly viewed as the primary mechanism underlying the reinforcing properties of cocaine (Ritz et al., 1987; Wise and Bozarth, 1987; Di Chiara, 2002), many important physiological effects of cocaine are resistant to DA receptor blockade (Kiritsy-Roy et al., 1990; Poon and Van Den Buuse, 1998; Tella and Goldberg, 1998). To test how DA receptor blockade affects cocaine-induced changes in NAc [glucose], we examined the effects of four cocaine injections after the rats were pretreated with a combination of selective D1-like (SCH23390) and D2-like (eticlopride) DA antagonists at doses that provide efficient blockade of DA transmission.

**Figure 5:**
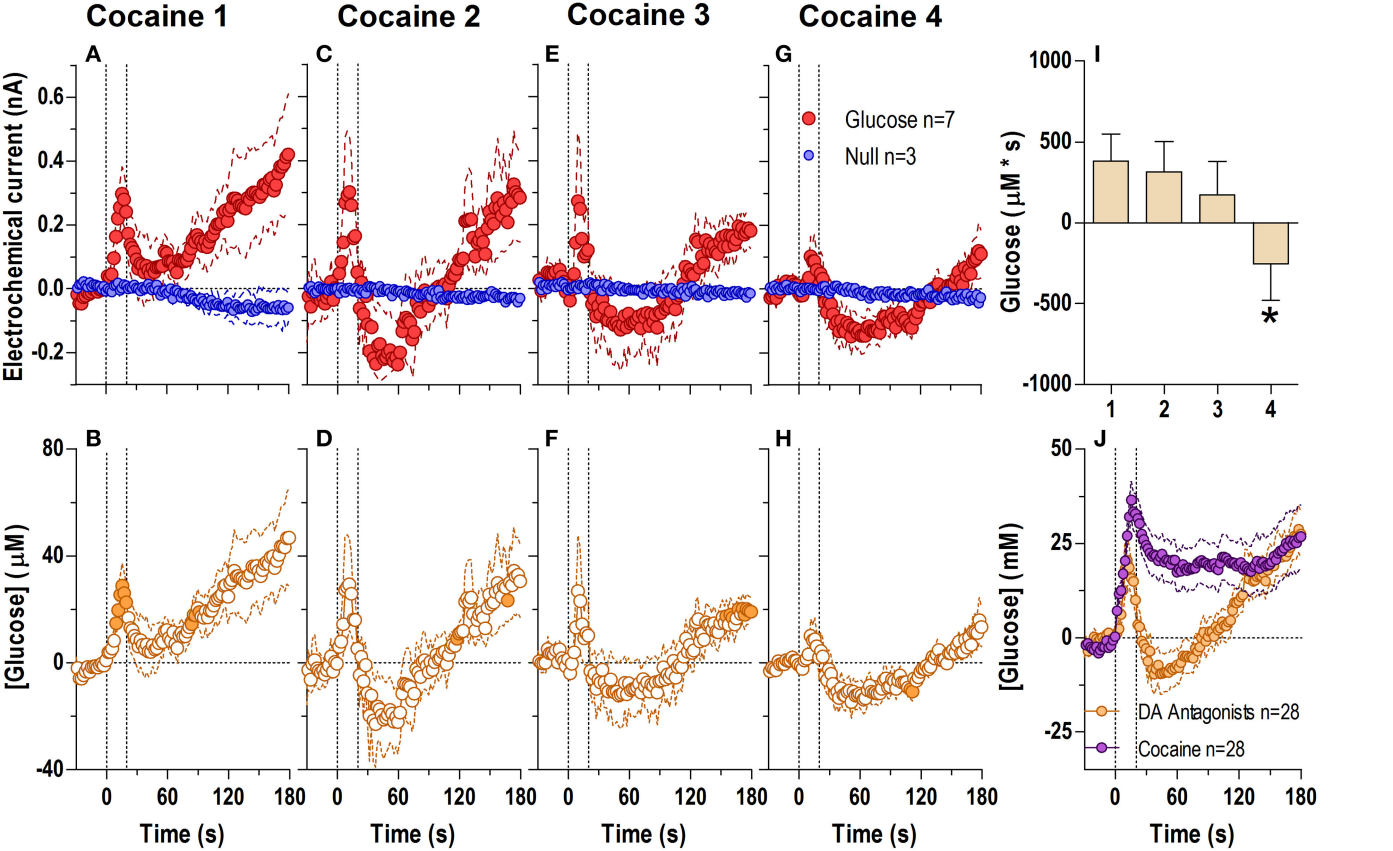
**Relative changes in NAc [glucose] induced by cocaine injections under conditions of full dopamine receptor blockade assessed at high temporal resolution (2-s bins)**. Top graphs **(A,C,E,G)** show mean ± SEM changes in relative currents (nA) detected by Glucose and Null sensors. Bottom graphs **(B,D,F,H)** show mean ± SEM changes in [glucose] (μM) as a difference between Glucose and Null sensors. Two vertical hatched lines (at 0 and 20) marked the onset and offset injection. Horizontal dotted lines show basal levels (= 0 nA and μM). The difference in current dynamics between Glucose and Null sensors was significant after each cocaine injection (**A** 1: Glucose/Null [25 s *F*_(1, 8)_ = 5.89], interaction [21 s *F*_(10, 80)_ = 2.24]; **C** 2, **E** 3, **G** 4: interaction [180 s, *F*_(90, 720)_ = 1.41, 1.28, 1.30, respectively] all *p* < 0.05. [Glucose] change was also significant for each cocaine injection [*F*_(6, 540)_ = 2.56, 3.53, 3.11, 3.62, respectively all *p* < 0.05] Concentration values significantly different from baseline (Fisher test) are shown as filled symbols. **(I)** shows significant differences in mean ± SEM glucose responses (area under curve) induced by four cocaine injections during DA receptor antagonism [*F*_(3, 18)_ = 6.62 *p* < 0.05]. Asterisk denotes significant (*p* < 0.05) differences between the 4th injection and all others (Fisher test). **(J)** shows significant differences in [glucose] between cocaine and cocaine-methiodide groups for the entire analysis window [Main effect: *F*_(1, 54)_ = 5.16, Treatment × Time interaction: *F*_(90, 4860)_ both *p* < 0.05] assessed for all drug injections.

When analyzed at a second-scale resolution (Figure 5), DA receptor blockade did not eliminate the first, rapid component of the cocaine-induced NAc glucose response (Figures 5A–H). Similar to untreated conditions, this immediate effect also showed progressive tolerance following repeated cocaine injections [Figure 5I; *F*_(3, 18)_ = 6.62 *p* < 0.05]. The overall glucose response to cocaine during DA receptor blockade was weaker than that with cocaine in untreated conditions and cocaine methiodide [effect of drug *F*_(2, 17)_ = 5.30 *p* < 0.05]. While the time-course of the cocaine-induced glucose rise was initially identical in both groups, the peak magnitude during DA antagonism was lower, and was followed by a more pronounced negative rebound (Figures 5D,F,H,I,J).

**Figure 6:**
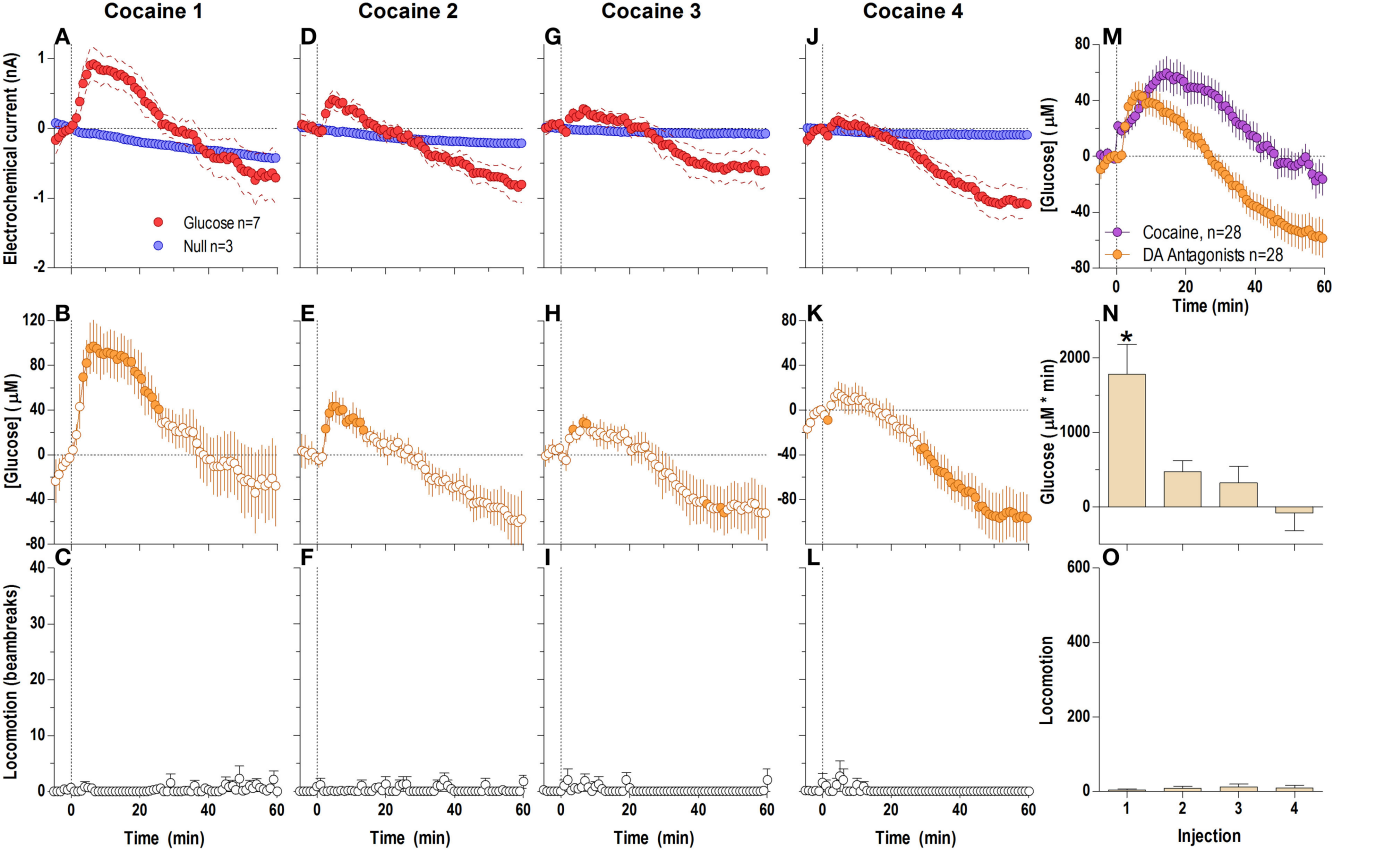
**Relative changes in NAc [glucose] induced by cocaine injections under full dopamine receptor antagonism assessed at low temporal resolution (1-min bins)**. Top graphs **(A,D,G,J)** show mean ± SEM changes in relative currents (nA) detected by Glucose and Null sensors. Middle graphs **(B,E,H,K)** show mean ± SEM changes in [glucose] (μM). Bottom graphs **(C,F,I,L)** show mean ± SEM changes in locomotion. Vertical hatched line (0 min) marked the onset of injection. Horizontal dotted lines denote basal levels (= 0 nA and μM). The difference in current dynamics between active and null sensors was significant (*p* < 0.05) for the entire analysis window (59.5 min) after each cocaine injection [Current × Time interaction: *F*_(60, 480)_ = 2.25, 2.83, 4.01, and 7.57, respectively, all *p* < 0.05] indicating a significant concentration change over the same time period for each cocaine injection [*F*_(6, 360)_ = 5.64, 7.10, 10.06, and 18.95, all *p* < 0.05] Concentration values significantly different from baseline (Fisher test) are shown as filled symbols. Right panel **(M)** compares mean ± SEM glucose responses induced by cocaine injections in control conditions and during DA receptor blockade [Main effect: *F*_(1, 54)_ = 9.32, Treatment × Time interaction *F*_(60, 3240)_ = 4.73, both *p* < 0.05]. **(N)** shows mean ± SEM [glucose] responses induced by cocaine injections during DA receptor antagonism assessed by area under the curve [*F*_(3, 18)_ = 10.53 *p* < 0.05]. Asterisk denotes significant differences between first and all other injections (Fisher test). **(O)** shows mean ± SEM locomotor responses (as area under curve); no significant between-injection changes were seen.

Surprisingly, DA receptor blockade that fully blocked the locomotor effects of cocaine (Figures 6C,F,I,L,O) did not block the second, tonic elevation of NAc glucose induced by cocaine (Figures 6A,B,D,E,G,H,J,K). The tonic rise in NAc [glucose] was greatest after the first cocaine injection (~100 μM), progressively decreased following subsequent injections, and was almost absent (~20 μM) after the last cocaine injection [Figure 6N; *F*_(3, 18)_ = 10.53, *p* < 0.05]. These changes were associated with a progressive enhancement of rebound-like decreases in NAc glucose, which were atypical to cocaine in untreated conditions. A between-group comparison (Figure 6M) revealed that during DA antagonism the rise in glucose was significantly less, its levels fell more strongly below the pre-injection baseline [effect of treatment: *F*_(1, 54)_ = 9.32 *p* < 0.05], and the response dynamics differed by a delayed onset and faster time to peak [Treatment × Time interaction *F*_(60, 3240)_ = 4.73 *p* < 0.05].

### Phasic increases in [glucose] induced by sensory stimuli

**Figure 7:**
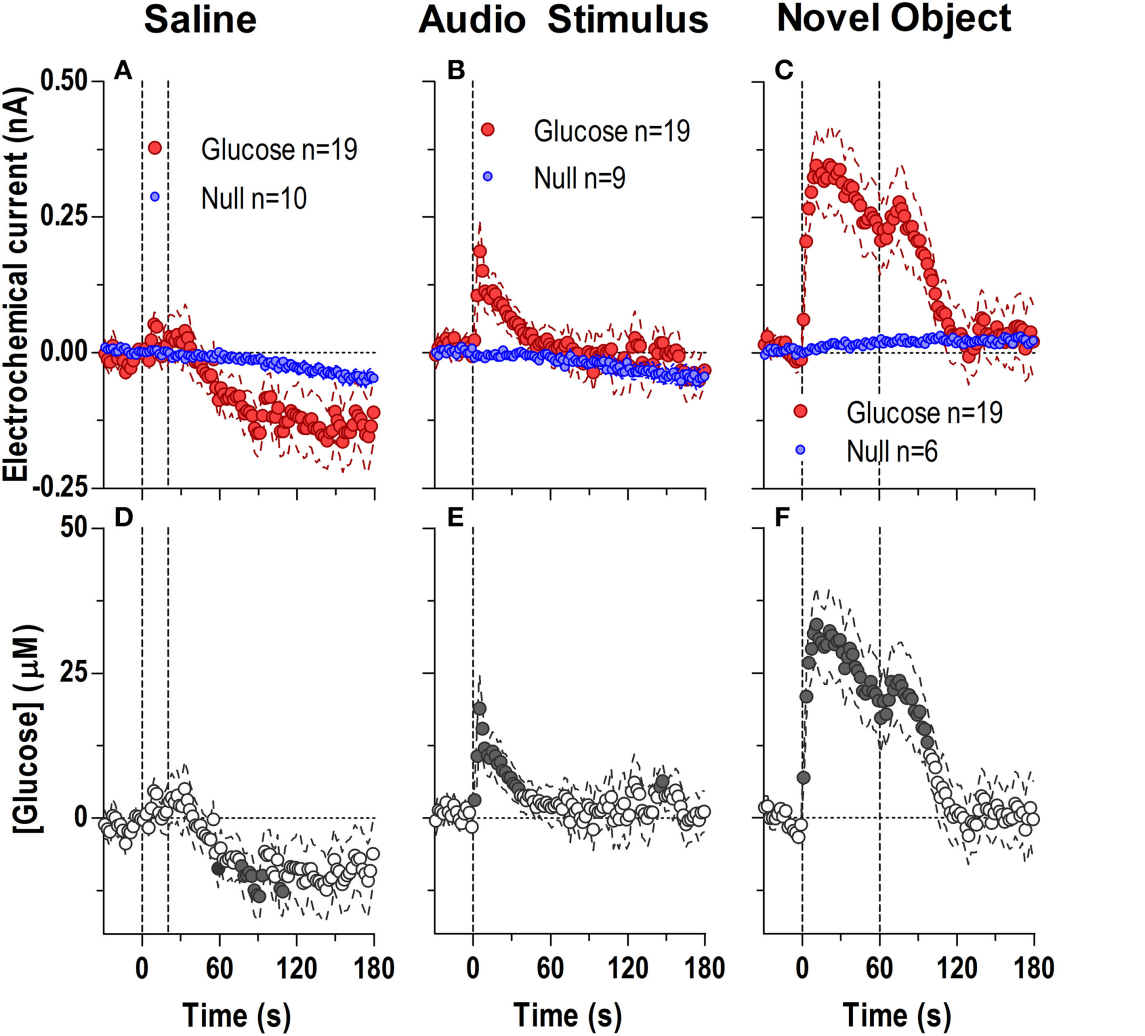
**Rapid changes in NAc [glucose] induced by saline injection (A,D) and exposure to a brief auditory stimulus (B,E) and a novel object (C,F)**. Top graphs show changes in Glucose and Null currents and bottom graphs show resulting changes in glucose concentration. Saline injections resulted in a significant difference in current dynamics (*p* < 0.05) for the entire analysis window [180 s; Interaction: *F*_(90, 2430)_ = 1.94 *p* < 0.05] indicating a significant decrease in glucose after the injection [*F*_(18, 1620)_ = 3.773 *p* < 0.05]. Concentration values significantly different from baseline (Fisher test) are shown as filled symbols. A brief audio stimulus and a novel object induced rapid and dynamic differences in Glucose and Null currents [Audio Stimulus, 129 s; Interaction *F*_(65, 1690)_ = 1.95; Novel object, 180 s; Interaction *F*_(90, 2070)_ = 4.34, both *p* < 0.05], revealing highly phasic glucose changes over the entire analysis window [Audio Stimulus, *F*_(18, 1620)_ = 2.41; Novel object, *F*_(18, 1620)_ = 14.85, both *p* < 0.05].

During each experiment, we also examined drug-free glucose responses induced by two sensory stimuli (a brief auditory stimulus and 1-min exposure to a novel object) and one or two injections of saline (Figure 7). The brief auditory stimulus induced a very rapid but short-lived difference in active and null currents [Figure 7B; 129 s, current × time: *F*_(65, 1690)_ = 1.95, *p* < 0.05] reflecting a rise of NAc [glucose] [Figure 7E: *F*_(18, 1620)_ = 2.41, *p* < 0.05] that became significant within the first 2–4 s after the stimulus onset. After peaking at 5–7 s (15–20 μM), [glucose] decreased gradually below the pre-stimulus baseline. A similarly rapid but stronger and more prolonged difference in active and null currents was found after the introduction of a novel object into the cage [Figure 7C; *F*_(90, 2070)_ = 4.34, *p* < 0.05]. This difference indicated a dynamic elevation in [glucose] [Figure 7F; *F*_(18, 1620)_ = 14.9 *p* < 0.05], which peaked at 10–20 s (~30 μM), remained elevated within the test, and showed an additional, weaker peak when the novel object was removed from the cage. In contrast to both sensory stimuli, stress- and cue-free iv injection of saline failed to induce significant changes in NAc glucose (Figures 7A,D).

### Results of histological verification of sensor locations

**Figure 8:**
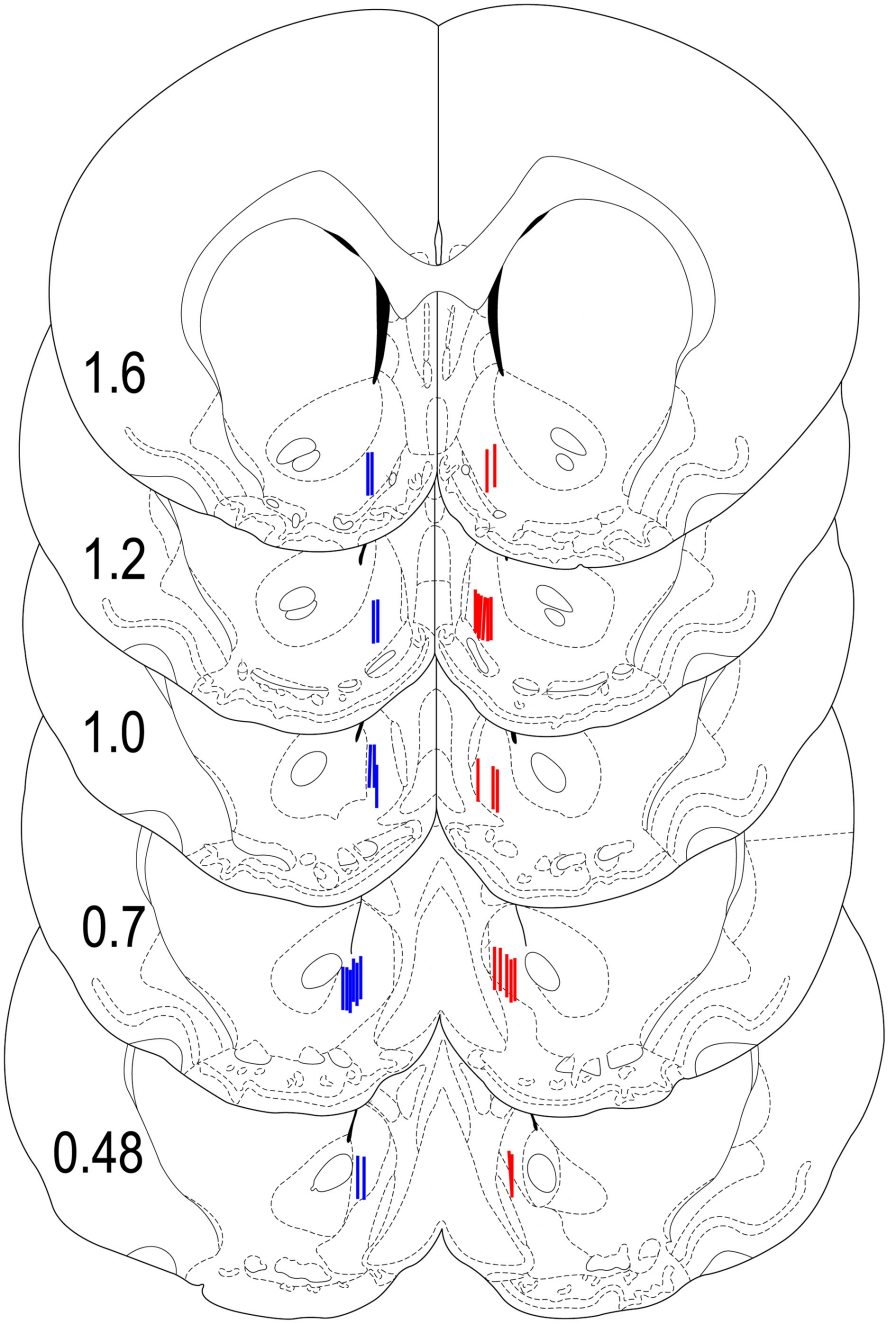
**Histological locations of electrochemical sensors**. Locations of the active area of Glucose (red) and Null (blue) sensors used in this study shown with the stereotaxic coordinates of Paxinos and Watson (1998). While the sensors were equally implanted in both sides of the brain, for clarity, glucose sensors are shown on the right and null sensors are shown on the left hemispheres.

Our previous studies suggest significant between-structure differences in glucose responses (Kiyatkin and Lenoir, 2012). Therefore, in this study it was critical to carefully examine the location of the recording sensors and exclude all cases where sensor tips were localized out of the target area. As can be seen in Figure 8, all sensors in rats included in our data set were closely localized within the NAc shell with relatively small dorso-ventral and anterior-posterior variability. Rats (*n* = 5), where the sensors were localized out of the target area were excluded from data analyses. While the number of rats in each group did not allow for a rigorous statistical evaluation of anterior-posterior differences in glucose responses, there were no evident differences along this axis of the NAc shell.

## Discussion

This study produced three novel findings. First, we showed that iv cocaine at a low, behaviorally active dose rapidly increases NAc extracellular [glucose], suggesting enhanced entry of this critical nutrient from the arterial blood to brain cells supporting their metabolic activity. Second, by using a BBB-impermeable cocaine analog, we demonstrated that the initial, rapid rise in [glucose] induced by cocaine is triggered via peripheral drug actions, possibly involving drug's action on afferents of sensory nerves and rapid neural transmission to the CNS via visceral sensory pathways. This peripherally driven neural mechanism was further supported by using simple and complex sensory stimuli that also induced equally rapid but transient increases in NAc glucose. Third, in addition to the initial phasic rise that was mimicked by sensory stimuli, cocaine also induced a larger tonic elevation in NAc glucose. This effect was absent with cocaine-methiodide, suggesting its dependence upon central actions of cocaine. Despite full blockade of locomotor activation, both components of cocaine-induced glucose response were only slightly inhibited by DA antagonists, indicating a major role of non-DA central mechanisms in their mediation. Finally, in contrast to previously reported sensitized neural responses to cocaine, both components of cocaine-induced NAc [glucose] increases underwent within-session tolerance, suggesting a possible experience-dependent dissociation of neural and metabolic effects of cocaine. Taken together, our results suggest that cocaine induces highly dynamic, experience-dependent changes in accumbal glucose inflow.

### Extracellular glucose and its physiological fluctuations

Unlike neurotransmitters and neuromodulators that are synthesized by brain cells and released into the extracellular space following neuronal activation, glucose enters the extracellular space from the arterial blood, where its concentration is 5–8-fold higher (Fellows and Boutelle, 1993; Silver and Erecinska, 1994; De Vries et al., 2003), and is continuously used by brain cells for their metabolic activity. Therefore, the extracellular [glucose] reflects the dynamic balance between two opposing variables: its entry into the brain tissue and its loss due to metabolic consumption. Although glucose biosensors provide an accurate picture of fluctuations in extracellular [glucose], this approach does not allow assessment of glucose consumption by brain cells. However, increases in [glucose] reliably indicate its inflow into the brain environment to satisfy metabolic demands of neural cells. Consistent with previous findings (Kiyatkin and Lenoir, 2012), NAc [glucose] rapidly increased following sensory stimulation, which is known to induce cortical EEG desynchronization (Kiyatkin and Smirnov, 2010), NAc glutamate release (Wakabayashi and Kiyatkin, 2012), and excitations of accumbal neurons (Kiyatkin and Brown, 2007). These findings as well as rapid increases in NAc [glucose] induced by local, intra-NAc glutamate microinjections (Kiyatkin and Lenoir, 2012), suggest neural activity as a critical trigger for rapid glucose entry into the extracellular space. While rapid, neural activity-driven increases in local cerebral blood flow (CBF) appears to be the primary mechanism that promotes inflow of glucose and oxygen to the active brain area (Attwell et al., 2010; Mergenthaler et al., 2013), intra-brain inflow of glucose and oxygen can also be enhanced as a consequence of metabolic activation due to the release of different metabolites and messengers from neurons and astrocytes that dilate brain arterioles and capillaries. This effect, however, is much slower but more prolonged. Finally, brain glucose levels could also slowly rise when blood glucose levels are rising (Kiyatkin and Wakabayashi, 2015). While this gradient-dependent mechanism is activated during glucose-drinking behavior (Wakabayashi et al., 2015) and systemic glucose administration, its contribution appears to be minimal under physiological conditions and after cocaine administrations.

### Peripheral triggering of the initial, ultra-fast rise in NAc [glucose] induced by iv cocaine

Iv cocaine passively administered to freely moving rats at a low, behaviorally active dose induced very rapid (~4-s latency) rise in NAc [glucose]. This initial response mimicked those induced by sensory stimuli and cocaine-methiodide, suggesting peripheral neural triggering. Since salient sensory stimuli, cocaine, and its BBB-impermeable analog all induce phasic excitations of most accumbal neurons (Kiyatkin and Rebec, 1996; Kiyatkin and Brown, 2007) as well as a rapid rise in NAc glutamate (Wakabayashi and Kiyatkin, 2012, 2014), this fast NAc glucose rise could result from its active, neural activity-driven entry into brain tissue. In addition to its known central actions on monoamine uptake, iv cocaine activates multiple ionic channels on the afferents of sensory nerves (Lee et al., 2005; Premkumar, 2005; Wu et al., 2006) that densely innervate blood vessels (Goder et al., 1993; Michaelis et al., 1994). This creates an ascending excitatory signal to the CNS, which is transmitted via visceral sensory pathways, resulting in generalized neural activation, involving the NAc shell.

While this ultra-fast rise in extracellular [glucose] could be viewed as surprising, many neural effects of iv cocaine are equally rapid, appearing within the injection duration and before the drug can physically reach brain tissue and act directly on its receptive substrates (Kiyatkin et al., 2000). In addition to EEG desynchronization, EMG activation, firing of accumbal neurons and NAc glutamate release that all appeared with 4–8-s latencies from the start of cocaine injection, an increase in arterial blood pressure peaks at ~10 s and significantly decays within 60 s post-injection (Poon and Van Den Buuse, 1998). Slightly larger, but still short onset latencies (10–20 s) are also found with cocaine-induced skin temperature decreases (Kiyatkin and Brown, 2007), another centrally mediated effect of cocaine that reflects peripheral vasoconstiction (Knuepfer and Branch, 1992). Similar to the ultra-fast rise in [glucose] in this study, all these neural and physiological effects are resistant to DA antagonism, which fully blocked cocaine-induced hyperlocomotion. It should be noted that DA antagonism slightly attenuated the rapid glucose rise, suggesting that only a small proportion of the normal glucose response to iv cocaine at this timescale is modulated by DA.

While the initial rapid NAc glucose rise induced by cocaine is obviously caused by active, neural activity-driven glucose entry, the mechanistic link between neural activity, local cerebral blood flow, and transporter-mediated facilitated diffusion of glucose via the BBB (Duelli and Kuschinsky, 2001; Barros et al., 2005) remains less clear. Since glucose influx into the extracellular space tightly correlate with changes in local CBF (Fellows and Boutelle, 1993) and iv cocaine rapidly increases CBF in both animals and humans (Stein and Fuller, 1993; Schmidt et al., 2006; Howell et al., 2010), this effect could be mediated in the NAc by local vasodilation, increases in local CBF, and accelerated glucose transport via the BBB.

### Slow cocaine-induced changes in NAc [glucose]: possible mechanisms

Cocaine also induced a second, tonic rise in NAc [glucose], which was the greatest (~100 μM or 10–12% over baseline) after the first cocaine injection and progressively decreased with subsequent injections. This decrease in responsiveness is in line with the well-known tolerance of cardiovascular effects of this drug (Smith et al., 1993; Lichtman et al., 1995; Tella et al., 1999; Wilson et al., 2000), suggesting the involvement of vascular mechanisms in its mediation, but it differs markedly from other neural, physiological and behavioral effects of cocaine, which are either stable or show experience-dependent sensitization (i.e., changes in DA and glutamate release; Addy et al., 2010; Wakabayashi and Kiyatkin, 2014). Although the absence of this effect with peripherally acting cocaine analog suggests that a direct central action of cocaine is essential in its mediation, it is more challenging to explain its mechanisms.

This slow component of NAc glucose rise could be a correlate of cocaine-induced metabolic activation that manifests in hyperlocomotion and increases in brain and body temperatures (Brown and Kiyatkin, 2006). In contrast to rapid neural effects, iv cocaine induced modest brain hyperthermia that occurred with ~60-s onset latencies, peaked at 20–25 min, and slowly decreased within 30–50 min, corresponding well with dynamics of the second, tonic NAc glucose rise. However, in contrast to the progressive decreases in slow glucose responses in this study, cocaine-induced brain temperature increases were relatively stable with a tendency for enhancement following repeated drug injections (Brown and Kiyatkin, 2006). While metabolic activation is an appealing explanation, peripherally acting cocaine-methiodide also increased NAc temperature (Brown and Kiyatkin, 2006), indicating metabolic brain activation, but did not induce tonic elevation in glucose levels in this structure. Surprisingly, tonic glucose responses were only slightly attenuated during pharmacological blockade of DA transmission, which fully blocks both motor-activating and hyperthermic effects of cocaine (Kiyatkin, 2008). Nevertheless, DA antagonists did influence the overall dynamics of this effect, indicating that DA is a minor contributor to the tonic component of cocaine-induced NAc glucose response. While the mechanisms underlying this slow effect of cocaine require further investigation, it could be at least in part related to slow elevations in blood glucose that occur after iv cocaine injection (Han et al., 1996). While reported only for larger doses (5 mg/kg, iv), a~20–30% rise in blood glucose (or 1–2 mM) detected in this study could potentially tonically increase NAc [glucose] within 100 μM.

Progressive tolerance of glucose responses also contrasts with previously reported changes in NAc glutamate (Wakabayashi and Kiyatkin, 2014). While the rapidity of the initial components of cocaine-induced glucose and glutamate responses and their tight correlation support a common link with local neural activation (Sibson et al., 1998; Attwell et al., 2010; Mergenthaler et al., 2013), in contrast to glucose, the NAc glutamate response progressively increased following repeated drug treatment. Interestingly, peripherally acting cocaine-methiodide, which induced only rapid, transient increases in NAc [glutamate] in drug-naive rats, induced robust glutamate responses in cocaine-experienced rats. While the mechanisms underlying the opposite experience-dependent changes in these two presumably tightly related neurochemical parameters (Attwell et al., 2010) remain unclear, a major source of slow fluctuations in extracellular glutamate could be astrocytes (Miele et al., 1996; Timmerman and Westerink, 1997; Kalivas, 2004; Vizi et al., 2010). Additionally, tolerance of slow glucose responses could be also related to its increased metabolic use or a decrease in its delivery from the blood, both processes where astrocytes play a prominent role (Attwell et al., 2010). Although the slower time course in both glucose and glutamate tonic changes suggests astrocyte or other glial cell involvement (Vizi et al., 2010; Howarth, 2014), additional work is needed to clarify these exact mechanisms.

### Extracellular glucose and brain metabolism

While monitoring of extracellular glucose shows real-time availability of this critical energetic substrate within the NAc, it is challenging to extend our findings with respect to cocaine's effects on brain metabolism and glucose consumption. Data obtained with deoxyglucose autoradiography and PET studies with radiolabeled glucose are controversial (see Introduction) due to differences in detection methodology, sampling periods, species, drug doses, brain structures, and the experimental conditions related to drug administration. Moreover, these data in fact provide a measure of glucose uptake but not its metabolism (Fillenz et al., 1999). While it is clear that the brain increases glucose and oxygen utilization upon activation (Sokoloff, 1999), real consumption of these substances appears to be less than their availability provided by rapid increases in local CBF and their enhanced entry into the extracellular space that prevents risky drops in these critical energetic substrates (Fox et al., 1988; Attwell et al., 2010; Mergenthaler et al., 2013).

While neuronal activation and subsequent slow changes in metabolites could be the primary factors determining functional hyperemia and enhanced entry of glucose and oxygen into the extracellular space, increases in CBF also serve to remove heat accumulated in brain tissue during metabolic activity. It is known that brain activity consumes large amounts of energy, all of which is eventually transformed into heat (Siesjo, 1978), resulting in relatively large (1–2°C) increases in brain temperature (Kiyatkin et al., 2002; Kiyatkin, 2010). In contrast to rapid changes in other neural parameters, cocaine-induced NAc temperature increases appeared much slower and peaked at ~20 min (Kiyatkin and Brown, 2005), paralleling tonic changes in glucose seen in this study. Therefore, intra-brain heat production is not only a valid measure of metabolic brain activation but appears to be a possible factor contributing to the known mis-match between the “excessive” rise in CBF with “over-delivery” of oxygen and glucose and the actual use of these metabolic substrates during functional brain activation (Fox et al., 1988).

## Conclusions and functional implications

Rapid increases in NAc extracellular [glucose] induced by iv cocaine result from active glucose entry from the peripheral circulation. This effect appears to be triggered by drug's actions on afferents of sensory nerves, resulting in neuronal activation, which is critical to induce an accelerated glucose entry into the NAc tissue. Therefore, the change in neuronal activity is not only the cause of multiple physiological and behavioral responses induced by cocaine, but also a factor that facilitates efficient delivery of required energetic resources such as glucose and oxygen to the areas of enhanced metabolic demand. The cocaine-induced increases in NAc [glucose] are within the range of physiological fluctuations (~50–100 μM or 7–15% of baseline) seen with natural arousing stimuli and they show rapid tolerance with repeated drug injections. While this pattern mimics that seen with vascular effects of cocaine, implying their involvement in rapid glucose transfer via the BBB, it contrasts to the sensitization of motor and some neural effects of cocaine. This mismatch between the increase in some neural responses and decrease in their metabolic supply could eventually trigger cocaine-induced neural, vascular, and behavioral abnormalities that are associated with cocaine addiction in humans (Volkow et al., 1991, 1993).

### Conflict of interest statement

The authors declare that the research was conducted in the absence of any commercial or financial relationships that could be construed as a potential conflict of interest.
